# Vaccination, Public Health and Health Communication: A Network of Connections to Tackle Global Challenges

**DOI:** 10.3390/vaccines13030245

**Published:** 2025-02-27

**Authors:** Antonella Arghittu, Giovanna Deiana, Marco Dettori, Paolo Castiglia

**Affiliations:** 1 Department of Medicine, Surgery and Pharmacy, University of Sassari, 07100 Sassari, Italy; madettori@uniss.it; 2 University Hospital of Sassari, 07100 Sassari, Italy; giovanna.deiana90@gmail.com

## 1. Background

Vaccination constitutes one of the most significant milestones in the history of Public Health. Throughout centuries of scientific and social advancement, vaccines have wiped out devastating diseases, curtailed the impact of epidemics and pandemics, and saved countless lives, establishing themselves as a cornerstone of global prevention. However, today’s health landscape presents increasingly complex challenges, driven by socio-cultural factors, global trends, environmental transformations and technological revolutions. Increased migration flows, climate change and urbanisation are altering epidemiological patterns and facilitating the spread of emerging pathogens. Simultaneously, inequalities in access to treatment and vaccine programmes are widening in fragile socio-economic contexts and low-income countries, exacerbating the health gap that exists from one region of the world to another. Against this backdrop, Vaccine Hesitancy has emerged as an insidious threat, capable of undermining the advances in vaccinology made in recent decades. Indeed, the topic of vaccination has become a contentious issue that goes beyond the medical sphere, and is now a subject of political, cultural, and social debate. Indeed, with the advent of Information and Communication Technologies (ICTs), we find ourselves immersed in a maelstrom of information that makes it difficult to distinguish between that which is scientifically sound and that which fuels a sense of confusion and fear. On the other hand, digital technologies, if properly harnessed, can act as catalysts to improve equity in access to vaccines, monitor vaccination coverage in real time, and raise awareness within various communities by breaking down geographical and cultural barriers. In line with this, Public Health has made numerous efforts to empower citizens to make informed health choices (e.g., voluntary adherence to vaccination or appropriate use of medicines). In this regard, the issue of vaccination is intertwined with that of antimicrobial resistance (AMR). Through its crucial role in the prevention of microbial infections, prophylactic vaccination reduces the need for medicines, particularly antibiotics, thus supporting the fight against one of the world’s most serious health emergencies. However, if vaccination is to have an effective, global, and sustainable health impact, it is essential to continue to diversify intervention strategies by age cohorts, risk categories, vulnerable individuals, and hard-to-reach groups, in tandem with exploiting the technological and informational innovations that can help counter the new determinants of Vaccine Hesitancy among the general population. The Special Issue entitled ‘New Insight in Vaccination and Public Health’ first published in October 2021 brought together international contributions on vaccine efficacy, strategies to reduce hesitancy, and the integration of Information and Communication Technologies (ICTs) in Public Health. Given the importance of such a topical subject, the second edition of this Special Issue aims at further expanding the research focus by exploring multidisciplinary solutions that address emerging challenges and strengthen the strategic role of vaccination in global health protection.

The history of vaccination is deeply intertwined with the advancement of Public Health, which is one of the most significant innovations in the history of medicine [1] (Contribution 1). Ever since the pioneering intuition of Edward Jenner, who introduced the smallpox vaccine in 1796, vaccines have significantly contributed to improving life expectancy and quality of life globally (Contribution 1) [2,3,4,5,6]. Currently, vaccines not only play a crucial role in preventing infectious diseases, but also act as a cornerstone of global strategies to address emerging health crises and improve the resilience of health systems [7,8,9]. However, like any scientific achievement, the advancement of vaccinology faces new challenges that emerge in response to the continuous evolution of biological, social, and environmental systems. Indeed, contemporary global trends such as international mobility, urbanisation, and climate change are reshaping the distribution and transmission of infectious diseases, with an impact that transcends national borders [10,11].

The growing threat of antimicrobial resistance (AMR) also underlines the urgency of effective preventive approaches. According to the World Health Organisation (WHO), AMR constitutes one of the major health emergencies of the 21st century, exacerbated by the inappropriate use of drugs [12,13,14]. In this context, preventive vaccination provides a crucial input in reducing the selective pressure on antibiotics and preventing resistant microbial infections. Evidence in the literature outlines that the implementation of targeted vaccination programmes can contribute significantly to mitigating the incidence of resistant infections, while generating economic benefits through reduced treatment-related healthcare costs [15,16,17,18]. In this context, the One Health approach, recognising the interdependence between human, animal, and environmental health, provides an indispensable operational framework to prevent and control such threats through coordinated and interdisciplinary interventions [19,20].

In addition, another critical aspect is the impact of digital technologies on immunisation [21,22,23]. Innovative tools such as big data analysis platforms and cloud computing infrastructures enable the precise identification of areas with low vaccination coverage as well as the prediction of potential outbreaks and the optimisation of resource allocation [21]. Similarly, m-health, through vaccination reminders and tracking systems, is a useful tool to improve population adherence and provide up-to-date information on ongoing vaccination campaigns [22,23].

Conversely, the digital environment has fuelled misinformation, making Vaccine Hesitancy an ever-present threat (Contribution 1) [24,25]. Defined by the WHO as a major Public Health problem, Vaccine Hesitancy is a complex and multifaceted issue, rooted in a combination of interconnected factors [26]. Historically, the ‘3C model’ of Vaccine Hesitancy, proposed in 2018 by WHO’s SAGE, offered a useful framework for understanding barriers to vaccine adherence [26,27,28]. Over the years, determinants such as pervasive digital misinformation, polarised public opinion, and precarious confidence in the institutions have fuelled a generalised distrust, thereby progressively hindering vaccination [26,29]. Moreover, recent research has introduced the concept of vaccination readiness, which refers to the willingness and readiness of individuals to be vaccinated, highlighting a dimension beyond traditional notions of hesitation. [30,31]. This led to the expansion of the ‘Cs’ paradigm, which is now structured in seven dimensions: Confidence, Complacency, Convenience, Calculation, Constraints, Collective Responsibility, and Conspiracy [32,33,34,35].

The above is even more worrying if one considers that scepticism towards vaccination is not only limited to the general public, but also extends to healthcare professionals, who, subjected to the same disinformation and social pressure, can sometimes find themselves in difficulty in their role as ambassadors of scientific confidence as well as vaccination facilitators [33]. Indeed, the credibility and communication skills of health professionals are fundamental in promoting public trust in vaccination programmes, although their effectiveness may be compromised without adequate support, education, and resources aimed at counteracting the pervasive influence of misinformation and scepticism [9].

Against this increasingly complex backdrop, it is essential to adopt a multidisciplinary scientific approach. To this end, current research needs to investigate and understand the social and cultural determinants of vaccine adherence in an ever-changing world, ensuring adaptable strategies for specific cohorts such as children, the elderly, immunocompromised individuals, and hard-to-reach marginalised groups [36].

Reflecting the interest sparked by the above-mentioned topics of debate, the first edition of the ‘New Insight in Vaccination and Public Health’ Special Issue collected 29 manuscripts (Contributions 1–29). The present work aims to explore the topics discussed within these articles in more detail, using a keyword network analysis. The goal is to provide a critical consideration that reinforces the main research questions raised, prompting innovative approaches to address emerging challenges and strengthen the role of vaccination as a cornerstone of global health.

## 2. Collection of Special Issue Articles

As of 31 October 2024, 2 Systematic Reviews, 1 Scoping Reviews, 6 Reviews, 2 Communications, 2 Editorials, and 16 Articles were selected following the peer review process. A total of 146 keywords were indicated by the 29 collected contributions. All manuscripts published and available online in open access form are listed in chronological order of publication in Table S1, including the following items: authorship, year, title, methodology, and keywords indicated in the abstract or body of the manuscript.

**Figure 1:**
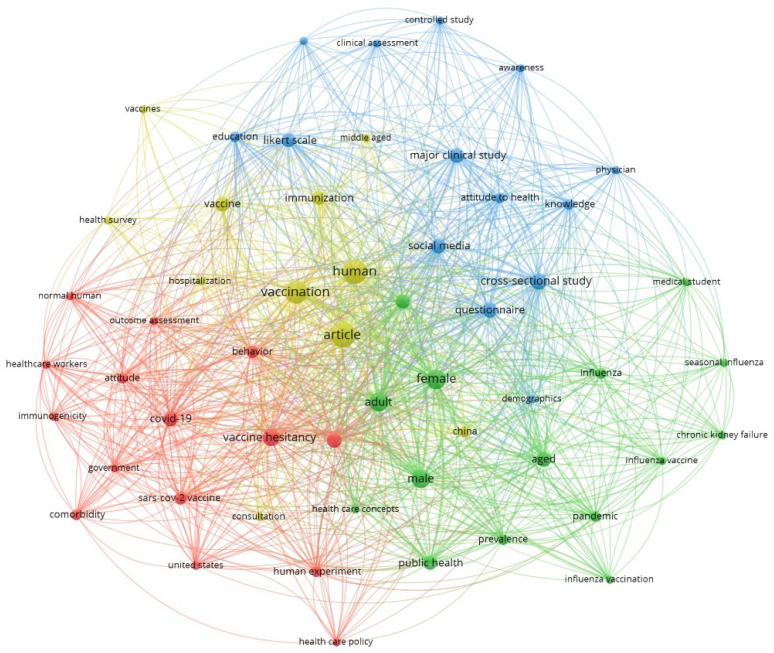
Network analysis of keywords in manuscripts published in the first edition of the Special Issue “New Insight in Vaccination and Public Health”.

In order to identify thematic relationships, emerging research areas, and interconnections between different scientific perspectives regarding the role of vaccination in Public Health, a keyword network analysis of the articles collected in the first edition of the Special Issue ‘New Insight in Vaccination and Public Health’ was conducted. To construct a word co-occurrence network, the keywords extracted from the published manuscripts were entered into a network analysis software (VOSviewer, available online: https://www.vosviewer.com/, accessed on 10 February 2025). The generated network made it possible to map connections between different topics, offering strategic guidance to address any upcoming challenges in the field of immunisation. This approach was used to translate a large amount of textual data into a visual model, facilitating the interpretation of the trends behind the scientific debate on vaccination covered in the first edition of this Special Issue (Figure 1).

## 3. Discussion

The network analysis conducted on the collection of articles published in the first edition of the Special Issue “New Insight in Vaccination and Public Health” made it possible to explore the conceptual connections emerging from the keywords in Contributions 1–29. By identifying central nodes, thematic clusters, and connections, it was possible to highlight interdisciplinary synergies and future challenges relating to the role of vaccination in global health promotion. At the centre of the network is the ‘vaccination’ node, which represents the convergence point around which the topics of cohort-specific vaccination (Contributions 2, 4, 8, 17, 18, 20, 23–27, and 29), digital communication (Contributions 7, 13, 15, and 21), and Vaccination Hesitancy (Contributions 3, 5, 6, 9–12, 14, 16, 22, and 28) revolve. These topics reflect the interdisciplinarity of the scientific debate on vaccination, as evidenced by the numerous thematic clusters and co-occurrences emerging from the network.

A first cluster closely related to Public Health highlights the imperativeness of planning and implementing vaccination strategies that respond promptly and effectively to health needs on a national and international scale. A clear example of this requirement can be seen in the numerous co-occurrences relating to the vaccination of specific population cohorts, such as the elderly, children, pregnant women, and vulnerable groups. In this regard, the reference to demographic variables such as ‘female’, ‘male’, ‘adult’, and ‘aged’ draws attention to the need for personalised approaches (Contributions 26, 27, and 29), also with a view to equity, highlighting the urgent need to address inequalities in access to vaccines by adopting inclusive policies. In this regard, various aspects of this cluster were explored in the first edition of this Special Issue, including the importance of conducting sero-prevalence surveys for varicella in pregnant women (Contribution 2), the analysis of gender and sex differences in response to COVID-19 vaccination (Contribution 4), knowledge and behaviour regarding HPV infection and adherence to vaccine programmes offered to adolescents (Contribution 8), the use of quantitative indicators in order to identify vaccine candidates (Contribution 17), the importance of preventive vaccination to promote healthy ageing (Contribution 18), the accessibility to vaccines against influenza (Contributions 20, 23, and 26), pneumococcal infection (Contribution 24), and Hepatitis B (Contribution 25). These insights show that planning the best vaccination strategies cannot disregard the adoption of flexible models capable of accommodating emerging priorities in a constantly changing global landscape.

Worthy of attention is the ‘social media’ node in the second cluster, which allows us to explore the issue of communication and disinformation management with particular attention to the role of digital platforms. By highlighting concepts such as ‘attitude to health’, ‘awareness’, and ‘knowledge’, the network replicated the complexity of the decision-making process, often exacerbated by what one reads online [37,38,39,40,41]. Digital platforms, with their ability to reach large demographics quickly, undoubtedly offer a unique opportunity to promote Public Health but, at the same time, pose a significant challenge in terms of countering misinformation (Contributions 7 and 15). This underpins the need to combat myths and misconceptions (Contribution 21) through a strategic approach that places an emphasis on the accuracy of information and its understanding (Contributions 1 and 19) [22,23]. In this regard, evidence in the literature indicates that accurate information accompanied by well-structured educational campaigns is a key means of increasing citizens’ knowledge and propensity to make informed health choices [22,23,24].

Misinformation management, moreover, is crucial if we are to combat the major Public Health problem that is Vaccine Hesitancy, defined by the WHO in 2019 as one of the top 10 threats to global health. This phenomenon, well highlighted in the third network cluster [26], is fuelled not only by long-standing scepticism towards vaccination but also by misinformation prevailing on the Web and negatively influencing perceptions, attitudes, and behavioural assumptions [22,23,24,26]. A distinctive element of this theme is the role of ‘healthcare workers’, connected via the Web to concepts such as ‘attitude’ and ‘behaviour’. Healthcare professionals, albeit active disseminators of correct information and promoters of a vaccination culture, are indeed themselves the recipients of information (Contributions 3, 5, and 9). As such, in a climate where misinformation can undermine even the experts’ beliefs, it is essential to ensure that health professionals receive constant, up-to-date, and targeted training so that they can act as an authoritative and reliable source for the general public [40,41].

This network analysis provided a complex and interconnected overview of the challenges related to vaccination, highlighting the need for an interdisciplinary approach that integrates research, health education, and public policy in coherence with training, project management, and outreach activities through target-specific communication campaigns. The future of global Public Health will depend on the ability to translate these interconnections into concrete actions through effective dialogue between all stakeholders.

With this in mind, the second edition of the Special Issue ‘New Insight in Vaccination and Public Health’ aims at identifying additional best practices of proven effectiveness in the field for the implementation of decisive strategies to achieve the coveted health goals. The objective is to stimulate new multidisciplinary research, capable of responding in an integrated and sustainable manner to the needs of an ever-changing society.

## Supplementary Materials

The following supporting information can be downloaded at: https://www.mdpi.com/article/10.3390/vaccines13030245/s1, Table S1: Description of the manuscripts accepted for the Special Issue “New Insight in Vaccination and Public Health” in chronological publishing order.
