# Supplementary material for: Vaccination, Public Health and Health Communication: A Network of Connections to Tackle Global Challenges
Source: Vaccines (Basel). 2025 Feb 27;13(3):245. doi: 10.3390/vaccines13030245 (PMC11945708; doi:10.3390/vaccines13030245)
Supplement: Supplementary file 1 [file vaccines-13-00245-s001.zip › vaccines-3499439-supplementary.pdf]

Table S1. Description of the manuscripts accepted for the Special Issue "New Insight in Vaccination and Public Health" in chronological publishing order.

Authorship	Year	Title	Methodology	Keyword
Lenis-Ballesteros V. et al. [1]	2021	Seroprevalence of Varicella in Pregnant Women and Newborns in a Region of Colombia	Cross-sectional study	Varicella; pregnant women; seroepidemiological studies; vaccine preventable disease; Colombia
Pal S. et al. [2]	2021	COVID-19 Vaccine Hesitancy and Attitude toward Booster Doses among US Healthcare Workers	Cross-sectional study	COVID-19; vaccine; booster; healthcare workers; United States
Heidari S. et al. [3]	2021	A Systematic Review of the Sex and Gender Reporting in COVID-19 Clinical Trials	Systematic Review	COVID-19; vaccines; sex; gender; SAGER Guidelines
Mackenzie L-J. et al. [4]	2022	Healthcare Practitioners Knowledge of Shoulder Injury Related to Vaccine Administration (SIRVA)	Survey	Shoulder Injuries Related to Vaccine Administration; SIRVA; iatrogenic; anatomical knowledge; immunisation; landmarking techniques
Motta M. [5]	2022	The Correlates & Public Health Consequences of Prospective Vaccine Hesitancy among Individuals Who Received COVID-19 Vaccine Boosters in the U.S.	Survey	COVID-19; vaccine hesitancy; public opinion; booster shots; health attitudes; health behavior
Aleksandric A. et al. [6]	2022	Spanish Facebook Posts as an Indicator of COVID-19 Vaccine Hesitancy in Texas	Descriptive analysis	Social media; sentiment; vaccine hesitancy; public health; interventions
Bencherit D. et al. [7]	2022	Knowledge and Awareness of Algerian Students about Cervical Cancer, HPV and HPV Vaccines: A Cross-Sectional Study	A Cross-Sectional Study	Cervical cancer; HPV infections; HPV vaccines; Algeria
Sallam M. et al. [8]	2022	The Role of Psychological Factors and Vaccine Conspiracy Beliefs in Influenza Vaccine Hesitancy and Uptake among Jordanian Healthcare Workers during the COVID-19 Pandemic	Cross-sectional study	Health professional; healthcare personnel; medicine practitioner; flu; barrier; vaccine behavior; vaccination intention; vaccine acceptance; seasonal influenza; influenza pandemic
Zemer V.S. et al. [9]	2022	Acceptance Rates of COVID-19 Vaccine Highlight the Need for Targeted Public Health Interventions	Observational study	COVID-19; COVID-19 vaccine; adults; comorbidity; vaccine hesitancy
Waszkiewicz P. et al. [10]	2022	Public Vaccination Reluctance: What Makes Us Change Our Minds? Results of A Longitudinal Cohort Survey	Survey	COVID-19; vaccines; vaccine hesitancy
Ma C. et al. [11]	2022	Prioritization of Vaccines for Inclusion into China's Expanded Program on Immunization: Evidence from Experts' Knowledge and Opinions	Delphi questionnaire	Vaccine; inclusion; Expanded Program on Immunization; Delphi; indicator system
Yin H. et al. [12]	2022	Factors Influencing the Knowledge Gap regarding Influenza and Influenza Vaccination in the Context of COVID-19 Pandemic: A Cross-Sectional Survey in China	Cross-sectional study	Influenza; influenza vaccination; knowledge gap; vaccine hesitancy; health communication; COVID-19 pandemic
Crăciun M.D. et al. [13]	2022	mRNA COVID-19 Vaccine Reactogenicity among Healthcare Workers: Results from an Active Survey in a Pediatric Hospital from Bucharest, January–February 2021	Survey	Active surveillance; COVID-19; mRNA vaccine; reactogenicity; healthcare workers
Olszowski R. et al. [14]	2022	A Social Network Analysis of Tweets Related to Mandatory COVID-19 Vaccination in Poland	Descriptive analysis	COVID-19; mandatory vaccination; vaccination hesitancy; social network analysis; social media; Twitter debate; Poland
Oduwole E.O. et al. [15]	2022	Overview of Tools and Measures Investigating Vaccine Hesitancy in a Ten Year Period: A Scoping Review	Review	Vaccine hesitancy; immunization; vaccination; tools; measures; scoping review
Marzouk M. et al. [16]	2022	Monitoring and Evaluation of National Vaccination Implementation: A Scoping Review of How Frameworks and Indicators Are Used in the Public Health Literature	Scoping Review	Vaccination; monitoring; evaluation; indicators; global health
Scognamiglio F. et al. [17]	2022	Vaccinations and Healthy Ageing: How to Rise to the Challenge Following a Life-Course Vaccination Approach	Review	Vaccination; healthy ageing; immune fitness; life-course vaccination
Castiglia P. et al [18]	2022	New Insight in Vaccination and Public Health: A Commentary from Special Issue Editors	Descriptive analysis	-

Arghittu A. et al [19]	2023	First Year of Special Issue "New Insights in Vaccination and Public Health": Opinions and Considerations	Descriptive analysis	-
Minardi V. et al [20]	2023	Influenza Vaccination Uptake and Prognostic Factors among Health Professionals in Italy: Results from the Nationwide Surveillance PASSI 2015–2018	Survey	Influenza vaccination; health personnel; health care worker; surveillance system; epidemiology; prevention; public health
Deiana G. et al [21]	2023	Artificial Intelligence and Public Health: Evaluating ChatGPT Responses to Vaccination Myths and Misconceptions	Survey	ChatGPT; vaccines; immunization; myths and misconceptions; public health; artificial intelligence
Eisenblaetter M. et al [22]	2023	Adaptation and Validation of a French Version of the Vaccination Attitudes Examination (VAX) Scale	Survey	Vaccine; vaccination attitudes; vaccination behaviours; vaccination intentions; scale development
Alshahrani S. et al [23]	2023	Prevalence and Predictors of Seasonal Influenza Vaccine Uptake in Saudi Arabia Post COVID-19: A Web-Based Online Cross-Sectional Study	Cross-Sectional study	Seasonal influenza; seasonal influenza vaccine; SIV; uptake; post COVID-19; prevalence; predictors; Saudi Arabia
Pi Z. et al. [24]	2023	Optimization of Elderly Influenza and Pneumococcal Immunization Programs in Beijing, China Using Health Economic Evaluations: A Modeling Study	Cost-effectiveness analyses	Cost-effectiveness; influenza; pneumococcal; vaccine
Stroffolini T. et al [25]	2023	Vaccination Campaign against Hepatitis B Virus in Italy: A History of Successful Achievements	Review	HBV; vaccination; Italy
Lindinger R. et al [26]	2023	Effect of the COVID-19 Pandemic on Paediatric Check-Ups and Vaccinations in Germany	Study Protcol	Paediatric check-ups; paediatric vaccinations; vaccines; COVID-19 pandemic; SARS-CoV-2; IQVIATM disease analyser; Germany
Koyuncu A. et al [27]	2023	The Use of Adaptive Sampling to Reach Disadvantaged Populations for Immunization Programs and Assessments: A Systematic Review	Systematic Review	Adaptive sampling; vaccine-preventable diseases (VPDs); immunizations; vaccines; hard-to-reach populations
Adamu A. et al [28]	2024	Mapping the Implementation Determinants of Second Dose Measles Vaccination in the World Health Organization African Region: A Rapid Review	Review	Measles-containing vaccines; childhood vaccination; WHO African Region; implementation determinants; consolidated framework for implementation research; primary health care
Stroffolini T. et al [29]	2024	Vaccination in Patients with Liver Cirrhosis: A Neglected Topic	Review	Cirrhosis; chronic liver disease; vaccines

References

1. Lenis-Ballesteros, V.; Ochoa, J.; Hincapié-Palacio, D.; León-Álvarez, A.; Vargas-Restrepo, F.; Ospina, M.C.; Buitrago-Giraldo, S.; Díaz, F.J.; Gonzalez-Ortíz, D. Seroprevalence of Varicella in Pregnant Women and Newborns in a Region of Colombia. Vaccines 2022, 10, 52. https://doi.org/10.3390/vaccines10010052
2. Pal, S.; Shekhar, R.; Kottewar, S.; Upadhyay, S.; Singh, M.; Pathak, D.; Kapuria, D.; Barrett, E.; Sheikh, A. COVID-19 Vaccine Hesitancy and Attitude toward Booster Doses among US Healthcare Workers. Vaccines 2021, 9(11), 1358; https://doi.org/10.3390/vaccines9111358
3. Heidari, S.; Palmer-Ross, A.; Goodman, T. A Systematic Review of the Sex and Gender Reporting in COVID-19 Clinical Trials. Vaccines 2021, 9(11), 1322; https://doi.org/10.3390/vaccines9111322
4. Mackenzie, L.; Bousie, J.; Newman, P.; Waghorn, J.; Cunningham, J.; Bushell, M. Healthcare Practitioners Knowledge of Shoulder Injury Related to Vaccine Administration (SIRVA). Vaccines 2022, 10(12), 1991; https://doi.org/10.3390/vaccines10121991
5. Motta, M. The Correlates & Public Health Consequences of Prospective Vaccine Hesitancy among Individuals Who Received COVID-19 Vaccine Boosters in the U.S.. Vaccines 2022, 10(11), 1791; https://doi.org/10.3390/vaccines10111791
6. Aleksandric, A.; Anderson, H.; Melcher, S.; Nilizadeh, S.; Wilson, G. Spanish Facebook Posts as an Indicator of COVID-19 Vaccine Hesitancy in Texas. Vaccines 2022, 10(10), 1713; https://doi.org/10.3390/vaccines10101713
7. Bencherit, D.; Kidar, R.; Otmani, S.; Sallam, M.; Samara, K.; Barqawi, H.; Lounis, M. Knowledge and Awareness of Algerian Students about Cervical Cancer, HPV and HPV

Vaccines: A Cross-Sectional Study. Vaccines 2022, 10(9), 1420; https://doi.org/10.3390/vaccines10091420
8. Sallam, M.; Ghazy, R.; Al-Salahat, K.; Al-Mahzoum, K.; AlHadidi, N.; Eid, H.; Kareem, N.; Al-Ajlouni, E.; Batarseh, R.; Ababneh, N.; Sallam, M.; Alsanafi, M.; Umakanthan, S.; Al-Tammemi, A.; Bakri, F.; Harapan, H.; Mahafzah, A.; Al Awaidy, S. The Role of Psychological Factors and Vaccine Conspiracy Beliefs in Influenza Vaccine Hesitancy and Uptake among Jordanian Healthcare Workers during the COVID-19 Pandemic. Vaccines 2022, 10(8), 1355; https://doi.org/10.3390/vaccines10081355
9. Shkalim Zemer, V.; Grossman, Z.; Cohen, H.; Hoshen, M.; Gerstein, M.; Yosef, N.; Cohen, M.; Ashkenazi, S. Acceptance Rates of COVID-19 Vaccine Highlight the Need for Targeted Public Health Interventions. Vaccines 2022, 10(8), 1167; https://doi.org/10.3390/vaccines10081167
10. Waszkiewicz, P.; Lewulis, P.; Górski, M.; Czarnecki, A.; Feleszko, W. Public Vaccination Reluctance: What Makes Us Change Our Minds? Results of A Longitudinal Cohort Survey. Vaccines 2022, 10(7), 1081; https://doi.org/10.3390/vaccines10071081
11. Ma, C.; Li, J.; Wang, N.; Wang, Y.; Song, Y.; Zeng, X.; Zheng, C.; An, Z.; Rodewald, L.; Yin, Z. Prioritization of Vaccines for Inclusion into China’s Expanded Program on Immunization: Evidence from Experts’ Knowledge and Opinions. Vaccines 2022, 10(7), 1010; https://doi.org/10.3390/vaccines10071010
12. Yin, H.; You, Q.; Wu, J.; Jin, L. Factors Influencing the Knowledge Gap regarding Influenza and Influenza Vaccination in the Context of COVID-19 Pandemic: A Cross-Sectional Survey in China. Vaccines 2022, 10(6), 957; https://doi.org/10.3390/vaccines10060957
13. Crăciun, M.; Nițescu, G.; Golumbeanu, M.; Tănase, A.; Pițigoi, D.; Săndulescu, O.; Crăciun, P.; Enciu, B.; Bălănescu, R.; Ulici, A. mRNA COVID-19 Vaccine Reactogenicity among Healthcare Workers: Results from an Active Survey in a Pediatric Hospital from Bucharest, January–February 2021. Vaccines 2022, 10(6), 836; https://doi.org/10.3390/vaccines10060836
14. Olszowski, R.; Zabdyr-Jamróz, M.; Baran, S.; Pięta, P.; Ahmed, W. A Social Network Analysis of Tweets Related to Mandatory COVID-19 Vaccination in Poland. Vaccines 2022, 10(5), 750; https://doi.org/10.3390/vaccines10050750
15. Oduwole, E.; Pienaar, E.; Mahomed, H.; Wiysonge, C. Overview of Tools and Measures Investigating Vaccine Hesitancy in a Ten Year Period: A Scoping Review. Vaccines 2022, 10(8), 1198; https://doi.org/10.3390/vaccines10081198
16. Marzouk, M.; Omar, M.; Sirison, K.; Ananthakrishnan, A.; Durrance-Bagale, A.; Pheerapanyawaranun, C.; Porncharoen, C.; Pimsarn, N.; Lam, S.; Ung, M.; Mougammadou Aribou, Z.; Dabak, S.; Isaranuwatchai, W.; Howard, N. Monitoring and Evaluation of National Vaccination Implementation: A Scoping Review of How Frameworks and Indicators Are Used in the Public Health Literature. Vaccines 2022, 10(4), 567; https://doi.org/10.3390/vaccines10040567
17. Scognamiglio, F.; Fantini, M.; Reno, C.; Montalti, M.; Di Valerio, Z.; Soldà, G.; Salussolia, A.; La Fauci, G.; Capodici, A.; Gori, D. Vaccinations and Healthy Ageing: How to Rise to the Challenge Following a Life-Course Vaccination Approach. Vaccines 2022, 10(3), 375; https://doi.org/10.3390/vaccines10030375
18. Castiglia, P.; Arghittu, A. New Insight in Vaccination and Public Health: A Commentary from Special Issue Editors. Vaccines 2022, 10, 183. https://doi.org/10.3390/vaccines10020183
19. Arghittu, A.; Dettori, M.; Castiglia, P. First Year of Special Issue “New Insights in Vaccination and Public Health”: Opinions and Considerations. Vaccines 2023, 11(3), 600; https://doi.org/10.3390/vaccines11030600
20. Minardi, V.; Gallo, R.; Possenti, V.; Contoli, B.; Di Fonzo, D.; D’Andrea, E.; Masocco, M. Influenza Vaccination Uptake and Prognostic Factors among Health Professionals in Italy:

Results from the Nationwide Surveillance PASSI 2015–2018. Vaccines 2023, 11(7), 1223; https://doi.org/10.3390/vaccines11071223
21. Deiana, G.; Dettori, M.; Arghittu, A.; Azara, A.; Gabutti, G.; Castiglia, P. Artificial Intelligence and Public Health: Evaluating ChatGPT Responses to Vaccination Myths and Misconceptions. Vaccines 2023, 11(7), 1217; https://doi.org/10.3390/vaccines11071217
22. Eisenblaetter, M.; Madiouni, C.; Laraki, Y.; Capdevielle, D.; Raffard, S. Adaptation and Validation of a French Version of the Vaccination Attitudes Examination (VAX) Scale. Vaccines 2023, 11(5), 1001; https://doi.org/10.3390/vaccines11051001
23. Alshahrani, S.; Zahrani, Y. Prevalence and Predictors of Seasonal Influenza Vaccine Uptake in Saudi Arabia Post COVID-19: A Web-Based Online Cross-Sectional Study. Vaccines 2023, 11(2), 353; https://doi.org/10.3390/vaccines11020353
24. Pi, Z.; Aoyagi, K.; Arima, K.; Wu, X.; Ye, Z.; Jiang, Y. Optimization of Elderly Influenza and Pneumococcal Immunization Programs in Beijing, China Using Health Economic Evaluations: A Modeling Study. Vaccines 2023, 11(1), 161; https://doi.org/10.3390/vaccines11010161
25. Stroffolini, T.; Stroffolini, G. Vaccination Campaign against Hepatitis B Virus in Italy: A History of Successful Achievements. Vaccines 2023, 11(10), 1531; https://doi.org/10.3390/vaccines11101531
26. Lindinger, R.; Richter, H.; Reuter, T.; Fischer, T. Effect of the COVID-19 Pandemic on Paediatric Check-Ups and Vaccinations in Germany. Vaccines 2023, 11(4), 720; https://doi.org/10.3390/vaccines11040720
27. Koyuncu, A.; Ishizumi, A.; Daniels, D.; Jalloh, M.; Wallace, A.; Prybylski, D. The Use of Adaptive Sampling to Reach Disadvantaged Populations for Immunization Programs and Assessments: A Systematic Review. Vaccines 2023, 11(2), 424; https://doi.org/10.3390/vaccines11020424
28. Adamu, A.; Jalo, R.; Masresha, B.; Ndwandwe, D.; Wiysonge, C. Mapping the Implementation Determinants of Second Dose Measles Vaccination in the World Health Organization African Region: A Rapid Review. Vaccines 2024, 12(8), 896; https://doi.org/10.3390/vaccines12080896
29. Stroffolini, T.; Stroffolini, G. Vaccination in Patients with Liver Cirrhosis: A Neglected Topic. Vaccines 2024, 12(7), 715; https://doi.org/10.3390/vaccines12070715
